# Use of Ergot of Rye

**Published:** 1846-01

**Authors:** J. Hall Davis


### SELECTIONS

FROM

### AMERICAN AND FOREIGN JOURNALS.

---

*Use of the Ergot of Rye.* By J. Hall Davis, M.D.—The efficacy of ergot of rye, as an excitant of uterine action, and a safe one under certain conditions, is now, I apprehend not doubted for a moment by those members of the profession who have had ample opportunities, from their connection with lying-in institutions, of sufficiently testing the merits of the drug. For my part, I can bear witness to its worth, when administered under proper precautions, and in no single instance have I found ill effects from its use. In improper cases, by virtue of its power it is undoubtedly capable of doing infinite mischief. The pains which it excites are continued, and much more powerful than the natural parturient throes; hence the violent respiratory efforts occasioned by it, would be apt to determine, in a plethoric subject, disturbance of the function of the brain; in a subject of heart disease, more surely an interruption of the functions of that organ. Under circumstances of a more than ordinary resistance to the agents of labor, from rigidity of the soft parts, or confinement of the pelvic space, the ill-judged exhibition of the medicine would be attended by an infinite risk of a rupture of the uterus and vagina. At a meeting of one of the medical societies in London, for the satisfaction of a member, and an unbeliever in the power of the drug, the uterus, just removed from a woman who had died of a rupture of the organ, was exhibited—that deplorable accident appearing to have resulted from the administration of the ergot of rye in an improper case; and there are not wanting other instances on record of the like result. It has been believed by some to destroy the child by a poisoning influence, but I

cannot discover any conclusive evidence in support of this assertion. Where the child has been still-born, it is my conviction that its death has arisen from an arrest of its circulation by inordinate pressure upon the body of the child and the funis; from the exhibition of the medicine in a case of more than ordinary resistance to the agents of labor, consequently under improper circumstances, or from some other cause quite accidental, and unconnected with its action.

The medicine is exhibited under various forms, in powder, with cold water, in infusion, decoction, and tincture.

In my own practice, I have preferred the infusion of the fresh powder in half-drachm doses, given every quarter of an hour. I have known a single dose suffice; but two, three, four, or five may be necessary. I do not exceed five or six doses; that amount failing more will be useless. Very frequently after the quantity necessary has been taken, the contents of the stomach are rejected; but then, generally, the medicine will have been retained sufficiently long to secure the object of its administration. The drug, having been given under a proper condition of the patient for its use, may be inefficient from the idiosyncrasy of the patient, as in the case of various other medicines, or from its being of a bad quality. It should present an ash-colored powder, possess a peculiar smell of its own, and form a dark violet infusion.

Upon the present occasion I shall confine my remarks to the exhibition of the ergot in labors at the full period of gestation, prior to the birth of the child, unattended by hæmorrhage; and, at a future opportunity, communicate a paper upon its application in tedious abortions, premature labor, upon the question as to its power of originating uterine action; and in uterine hæmorrhage, under certain circumstances, at all periods of pregnancy and labor; as also in the instance of morbid deposits, hydatids, moles, &c., in the uterine cavity. The following are the conditions required for its safe employment in tedious labor at the full period of gestation.

1. The soft parts lax, and free from heat.

2. With rare exceptions the orifice of the uterus should be fully or nearly fully, dilated, and always dilatable.

3. The pelvic space should present the average good or standard dimensions.

4. In head presentations only; in breech and footling cases it is objectionable, on account of the very gradual descent of the presenting parts required for the subsequent passage of the head, without risk to the cord; in transverse presentations it is obviously improper.

5. The head should be in an average good position, and not be impacted.

6. The inertia of the womb should not have for its source a state of plethora, to be recognized by the obvious indications of that condition.

7. The absence of any source of irritation in any other organ capable of disturbing the parturient function by reflex action, should be first ascertained, as of fæcal accumulations, urine in the bladder, crude ingesta, to be met by their obvious indications of treatment.

8. The uterine inertia should be ascertained not to depend upon disturbance of the nervous function from loss of rest, or from depressing emotions, as great anxiety, painful anticipation, want of confidence in the attendant, &c.

9. There should not be present any cause of distention of the womb beyond its power of acting to advantage, as by excessive quantity of liquor amnii, twins.

10. It would not be indicated when the inertia, arose from constitutional weakness, to be preferably met by appropriate constitutional measures.

11. It is very rarely advisable to employ the ergot in the labors of those who are parturient for the first time; for myself, I have never given it in primiparæ; much time, and a slower action of the parturient powers, being required, than is consistent with the effects of the medicine.

In short, this remedy being indicated on account of inertia of the womb, arising from the effect of previous distentions of the organ, or from no ascertainable cause, there should be, for its safe exhibition, a natural presentation, a pelvis of ample dimensions, a free passage in other respects, the soft parts relaxed, and dilatable, nothing wanting, in a word, but efficient action of the uterus to finish the labor.—*London Lancet.*

